# Passive movement therapy in patients with moderate to severe paratonia; study protocol of a randomised clinical trial (ISRCTN43069940)

**DOI:** 10.1186/1471-2318-7-30

**Published:** 2007-12-19

**Authors:** Johannes SM Hobbelen, Frans RJ Verhey, Jacobus HJ Bor, Rob A de Bie, Raymond TCM Koopmans

**Affiliations:** 1 Physiotherapy Research Vitalis WoonZorg groep Eindhoven, The Netherlands; 2 School for Public Health and Primary Care (CAPHRI), Maastricht University PO Box 616, 6200 MD Maastricht, The Netherlands; 3 University Hospital of Maastricht/Alzheimer Centre Limburg PO Box 5800, 6202 AZ Maastricht, The Netherlands; 4 Department of Nursing Home Medicine Radboud University Nijmegen Medical Centre P.O. Box 9101, 229 VPG 6500 HB Nijmegen, The Netherlands; 5 Department of Epidemiology, Maastricht University PO Box 616, 6200 MD Maastricht, The Netherlands

## Abstract

**Background:**

Paratonia, a form of hypertonia, is associated with loss of mobility and with the development of contractures especially in the late stages of the dementia.

Passive movement therapy (PMT) currently is the main physiotherapeutic intervention. General doubt about the beneficial effects of this widely used therapy necessitates a randomised clinical trial (RCT) to study the efficacy of PMT on the severity of paratonia and on the improvement of daily care.

**Methods/Design:**

A RCT with a 4-week follow-up period. Patients with dementia (according to the DSM-IV-TR Criteria) and moderate to severe paratonia are included in the study after proxy consent. By means of computerised and concealed block randomisation (block-size of 4) patients are included in one of two groups. The first group receives PMT, the second group receives usual care without PMT. PMT is given according to a protocol by physical therapist three times a week for four weeks in a row. The severity of paratonia (Modified Ashworth scale), the severity of the dementia (Global Deterioration Scale), the clinical improvement (Clinical Global Impressions), the difficulty in daily care (Patient Specific Complaints) and the experienced pain in daily care of the participant (PACSLAC-D) is assessed by assessors blind to treatment allocation at baseline, after 6 and 12 treatments.

Success of the intervention is defined as a significant increase of decline on the modified Ashworth scale. The 'proportion of change' in two and four weeks time on this scale will be analysed. Also a multiple logistic regression analysis using declined/not declined criteria as dependent variable with correction for relevant confounders (e.g. stage of dementia, medication, co-morbidity) will be used.

**Discussion:**

This study is the first RCT of this size to gain further insight on the effect of passive movement therapy on the severity of paratonia.

**Trial registration:**

Current Controlled Trials ISRCTN43069940

## Background

Paratonia is one of the motor problems seen in persons with dementia and was first described by Dupré in 1910 as the inability to relax muscles in combination with a mental disorder [1]. Paratonia is hypothesized to develop centrally but to exert an effect on peripheral biomechanics. Especially in the late stages of dementia it is associated with loss of mobility and with the development of contractures [2-5]. Thus, paratonie has a negative impact on the quality of life and can result in problems with washing and dressing

The prevalence of paratonia ranges from 5% in the early stages of dementia to 100% in the advanced stages [2,5]. We found an estimated prevalence of paratonia of approximately 80% in a group of Dutch nursing home patients with dementia [6]. Passive movement therapy (PMT) is a therapeutic intervention designed to increase the passive extensibility of muscles, ligaments and collagen in order to achieve maximal joint range of motion [7,8]. A recent NIVEL report revealed that PMT is with 28.2% one of the main physiotherapeutic interventions in Dutch nursing homes, with an average duration of 30 minutes per patient per week [9]. This therapy is generally believed to be effective in patients with paratonia [10,11]. Professional workers claim that this therapy, if given shortly before washing and bathing, facilitates the care for the patients, due to improved range of motion of affected limbs. Investigations in other populations, e.g., patients with spasticity and contractures show a temporal effect or a so-called elastic deformation due to the visco-elastic properties in all tissues [7]. However, after 20 to 30 minutes, joint range of motion returns to the starting values. Plastic deformation, or a permanent effect, is only possible if the patient can actively use the gained mobility[7,8]. Furthermore, animal studies indicate that when activated muscles fibres are stretched, which is the case with PMT in paratonia, older tissues are more susceptible to injury on sarcomere level [12]. Given the fact that these frail patients, who often show signs of discomfort during the treatment, are prone to injury and are not able to actively use regained mobility PMT is controversial. Nonetheless, maybe because of a lack of alternatives, or because of pressure of concerned relatives, physicians and physiotherapist start PMT. A pilot study in 15 patients with paratonia confirmed that PMT had positive short term effects, but trend analyses of long term effects showed that after 3 weeks hypertonia increased slightly in 30% in the PMT group in comparison with 10% in the control groups, indicating a possible association with muscle fibre injuries [3]. We feel this is a relevant finding, however, the small sample size of this pilot study, and the lack of a clear operational definition of paratonia did not allow for firm conclusions concerning the efficacy of PMT.

Before designing a new trial with sufficient power we therefore initiated a Delphi procedure with known experts in the field to achieve a new consensus definition of paratonia. After four Delphi-rounds, the experts agreed on 7 criteria for operational defining paratonia (see below) [13]. Consequently these criteria have been assessed on validity and reliability in a 3-phase cross-sectional study in which this definition was tailored even further and in which we developed an assessment instrument (the Paratonia Assessment Instrument, PAI) for an instant diagnosis of paratonia, thus providing researchers an operational tool for future trials on paratonia [6].

*"Paratonia is a form of hypertonia with an involuntary variable resistance during passive movement. The nature of paratonia may change with progression of the dementing illness (e.g. active assistance (Mitgehen) is more common early in the course of degenerative dementias, whilst active resistance is more common later in the course of the disease). The degree of resistance varies depending on the speed of movement (e.g. a low resistance to slow movement and a high resistance to fast movement). Paratonia increases with progression of dementia. Furthermore, the resistance to passive movement is in any direction and there is no clasp-knife phenomenon." The resistance must be felt in either two directions in one limb or in two different limbs*.

We designed a randomized clinical trial in order to answer three research questions; first, is passive movement therapy an effective intervention on the severity of paratonia in comparison with usual care without passive movement therapy?

Second, is passive movement therapy an effective intervention for improvement of daily care? And finally, does PMT reduce pain during daily care in patients with moderate to severe paratonia.

## Methods

To answer these 3 research questions we use a randomised clinical trial with 4 weeks of follow-up.

### Patients

The study population consists of patients with dementia (according to the DSM-IV-TR Criteria) and established paratonia according to the Paratonia Assessment Instrument (PAI). The PAI is a construct of five criteria derived from the definition, representing distinct elements of the clinical manifestation of paratonia [6]. The resistance felt during passive movement due to paratonia has to be at least in one of the limbs more marked i.e. a score of 2 or more on the modified Ashworth scale. Possible participants will be identified by trained personnel of participating nursing homes. After identification of participants the researcher checks if the patient is eligible for the study and if so will contact the legal representative(s) of the patient by sending an information leaflet about the study. Patients are only included after proxy consent. Because patients are included after proxy consent we will exclude participants who indicate during the trial in any way, verbally or nonverbally, not to approve of participation. Patients with an unstable disease, as judged by the nursing home physician, such as progressive malignant cancer or other diseases with an obvious progressive negative effect on the motor function or health status are excluded as well as patients who received passive movement therapy within a period of 4 weeks prior to admission or who receive typical or a-typical antipsychotics.

### Interventions

Patients are included in one of two groups after computerised and concealed block randomisation (block size of four). The first group receives usual care with PMT, the second group receives usual care without PMT. Usual care generally consists of grooming and dressing with slow and gentle movements by trained nurses. Some of the participants wear especially designed clothing that enables the nurses to dress patients more easy while the patient is lying in bed or sitting in a wheelchair. Most participants use cushions, mostly manufactured on demand, for a stable position in bed and sit during the day time in comfortable wheelchairs.

PMT is provided in a standardized way. During the first part of passive movement the therapist moves slowly the affected limbs, with the emphasis on lowering the resistance. After this, the therapist tries to reach the end range of motion and possibly stretches the structures very lightly without causing pain. The patients are positioned comfortably supine in bed while the therapist starts PMT with the left arm moving it in flexion and extension (up and down). Subsequently PMT is performed on the right arm, left leg and finally the right leg. The duration of PMT is approximately 20 minutes per patient per session. The treatment group receives PMT, between 8 a.m. and 10 a.m., shortly before being washed and dressed by nursing staff, three times a week for four weeks in a row. In order to safeguard blinding of the assessors for treatment allocation, the control group receives a placebo treatment in the same frequency and in the same time frame. The patients of the control group are positioned comfortably supine in bed after which the therapist stays in the room for approximately 20 minutes. On every treatment day a special signboard on the participant's bedroom door indicates that research is going on advising nursing staff to delay their activities with the participant and not disturb the treatment session.

### Outcome measures

The Modified Ashworth scale is the primary outcome measure and tested with an acceptable reliability to assess the severity of paratonia (intrarater reliability; Kendall's Tb 0.62–0.80 and interrater reliability; Kendall's Tb 0.72–0.77) [14]. It is a 5 point scale ranging from 0 to 4, in which 0 = no resistance to passive movement, 1 = slight resistance during passive movement, 2 = more marked resistance to passive movement, 3 = considerable resistance to passive movement, 4 = severe resistance, passive movement is impossible. Severity of paratonia will be assessed by assessors blinded to treatment allocation at baseline one day prior to treatment start, one day after treatment 6 (after 2 weeks) and one day after treatment 12 (after 4 weeks) between 8 a.m. and 10 a.m. before washing and dressing by nursing staff.

To assess the severity of paratonia all four limbs will be passively moved in flexion and extension with the participant in a comfortable position supine in bed.

As secondary outcome measures we assess The Pain Assessment Checklist for Seniors with Limited Ability to Communicate (PACSLAC-D) [15,16], to assess a decrease of pain as a possible side effect of PMT The PACSLAC-D is an observational assessment instrument that lists 24 items divided in three categories; nonverbal facial signs (10 items), total resistance (6 items) and emotional state (8 items). This version, a reduction of the original 60 item PACSLAC scale had high levels of internal consistency for the complete scale (Cronbach's alpha range 0.82–0.86) and for all subscales (alpha range 0.72–0.82) [17].

An independent observer will assess the PACSLAC-D in both groups by a 5-minute observation of washing and dressing within an hour after the first, sixth and the twelfth treatment session. Another secondary outcome measure is the Clinical Global Impressions scale (CGI) to assess clinical change. With this CGI, especially appointed nurses, who are blinded for treatment allocation, compare the participant with all other patients with paratonia on their ward on a 7 point scale from normal to most severe and rate after 2 and 4 weeks the global improvement also on a 7 point scale from very much improved to very much worse. Finally, the modified "Patient Specific Complaint" (PSC) assessment is used, in which the nurses are asked to address the 3 most difficult items in daily care and rate these items on a visual analogue scale of 100 mm, with at the extreme ends "no trouble at all" and "impossible". The frequencies of all assessments are illustrated in Table 1.

**Table 1:** Assessment schedule

**Assessment scale *(Assessed by)***	**T0**	**T1**	**T2**
**Modified Ashworth *(Assessor, blind for treatment allocation)***	One day prior to treatment start	One day after treatment 6	One day after treatment 12
**PACSLAC *(independent assessor, blind for treatment allocation)***	on the day of the first treatment	On the day of the sixth treatment	On the day of the twelfth treatment
**CGI and PSC *(Nurse, blind for treatment allocation)***	One day prior to treatment start	One day after treatment 6	One day after treatment 12
**GDS *(Nurse)***	Within a week before trial start		

At baseline we will register demographic information and relevant variables such as age, sex, use of medication, type of dementia and severity of the dementia. Severity of dementia is assessed with the Global Deterioration Scale which consists of seven stages of cognitive decline in which stage 1 = no cognitive decline, level 2 = very mild cognitive decline, level 3 = mild cognitive decline, level 4 = moderate cognitive decline, level 5 = moderately severe, level 6 = severe and level 7 = very severe cognitive decline [18]. Psychoactive medications will be classified using the Anatomical Therapeutical Chemical-classification and grouped into antipsychotics, anxiolytics, hypnotics/sedatives, antidepressants, anti-epileptics and miscellaneous (e.g. cholinesterase inhibitors) [19].

### Sample size

The Modified Ashworth Scale is our primary outcome measure. A trend analysis of the pilot study data showed a worsening of paratonia in 30% of the group with PMT and in 10%, possibly due to natural course, of the group with usual care without PMT [3]. We consider this effect as clinical important. With an alpha of 0.05 and a power of 80% a sample size of 69 patients per group (taking into account a drop-out percentage of 10%) is needed to detect this effect.

### Analysis

All data will be analysed with SPSS 15.0. The Modified Ashworth scale, an ordinal 5-point scale, will be measured at 3 times, at baseline (T0) after 2 weeks (T1) and after 4 weeks (T2). Our premise in this study is that PMT causes an accelerated worsening of paratonia over 4 weeks time. Success of the intervention is defined as a significant (p < 0.05) difference between the proportion of change on the modified Ashworth scale in the two groups.

To determine any development or change over time of The Modified Ashworth score in the two groups the Stuart-Maxwell statistic will be used.

The difference in proportion of change between the two groups will be calculated and tested for significance [20]. However controlling for relevant confounders e.g. age, sex, severity and type of dementia is not possible with this analysis. Therefore a multiple logistic regression analysis will be performed on the dichotomised outcome measure, "declined" and "stable/improved", of the difference on T0 and T2 on The Modified Ashworth score.

Missing data will be assumed to be missing at random. The Last Observation Carried Forward method will be used in those cases with no last measurement (T2) yet with valid data from the second assessment (T1). Analyses will be carried out according to the intention to treat principle.

### Study timeline

The estimated project time is 18 months. The project will start with an extensive training of relevant staff of all participating nursing homes. The training period will take two months. After this training the inclusion period will start and will run for twelve months. Analysis of data and writing of the report is estimated to take 4 months.

The study has been approved by the local ethical committee CMO nr. 2006/1567, ABR file nr. NL13777.091.06.

The investigator will notify the accredited local ethical committee of the end of the study within a period of 8 weeks. The end of the study is defined as the last patient's last follow-up measurement. Within one year after the end of the study the investigator will submit a final study report with the results, including any publication/abstracts of the study to the accredited local ethical committee. The investigators are not restricted in any way to publish the results of the study.

## Discussion

This study is the first RCT of this size to gain further insight on the effect of passive movement therapy on the severity of paratonia.

### Ethical aspects

PMT is a very common physiotherapeutic intervention in patients with moderate to severe paratonia. Although it is controversial and possibly harmful for these frail elderly, in lack of evidence based alternative interventions, most therapists perform PMT. For this reason we designed this RCT in a very pragmatic way, close to daily practice bounded by ethical aspects limiting the time frame of 4 weeks per participant and using assessment tools that are valid and reliable but above all with a minimal burden for the frail participants. Although we realise that further assessments after the end of the treatment period could have given more insight in the duration of the effect of PMT, the frailty of the participants prevails over the aims of research. For the same reason we decided to use no invasive methods, such as muscle biopsy to assess possible tissue damage.

### Bias

With the use of the consensus definition a homogeneous population should be guaranteed although research in the more severe cases of paratonia indicates that if the resistance in affected limbs is very high the assessor is not able to accelerate the movement and adequate differentiation with Parkinsonian (lead pipe) rigidity becomes impossible [6]. Furthermore, we did not specify the type of dementia in the inclusion criteria. At this moment we know that in the advanced stages of the disease all patients have paratonia. However, it is unclear if there are any dissimilarities in the development and severity of paratonia in different types of dementia. Therefore paratonia, diagnosed in different dementias, could react inconsistently to PMT and although we take this into account in our analysis this uncertainty could be a potential source of bias.

With this study, we hope to clarify the effect of passive movement therapy on moderate to severe paratonia and hopefully gain some scientific basis for the use of this treatment or to abandon PMT in this population. The controversy between the hypothesis of the research team that PMT is possibly harmful and the assumption of nurses and relatives that it is beneficial, necessitates a thorough organisation for keeping all assessors blind for treatment allocation. Therefore randomisation is done in a block size randomisation system with block size of four. For each participating nursing home a new sequence is calculated at the faculty of Epidemiology of the Maastricht University. The blinding of the assessors can be compromised by an incidental verbal hint of one of the participants although most of the participants are in the advanced stage of dementia and lost there capacity of speech.

### validity

The research will be conducted in four different nursing homes, that will enhance extrapolation of study findings. However, due to the fact that at least 4 different geriatric physical therapists will perform PMT and at least two different assessors of the severity of paratonia will participate, the internal validity may become compromised. A protocollized way of performing PMT and an extensive training of all therapists and assessors involved should ensure internal validity of this study and reliability of assessed outcomes.

The results of this randomised controlled trial will be published in a scientific journal and will be used for recommendations in the guideline of geriatric physical therapy and implemented in current physical therapy practice. This guideline will be developed according to method of clinical practice guideline development of the Royal Dutch Physiotherapy Association (KNGF) [21].

## Competing interests

The authors declare that they have no competing interests. The investigators are not restricted in any way to publish the results of the study

## Authors' contributions

JH designed the study and drafted the manuscript, FV participated in the design of the study and helped to draft the manuscript, JB helped writing the statistical paragraph of the protocol, RB participated in the design of the study and helped to draft the manuscript, RK participated in the design of the study and to draft the manuscript. All authors have given final approval of the version to be published.

## Pre-publication history

The pre-publication history for this paper can be accessed here:


